# Real‐World Therapeutic Hypothermia for Neonatal HIE: Neurodevelopmental Outcomes and Predictors

**DOI:** 10.1111/apa.70186

**Published:** 2025-06-18

**Authors:** Luca Bedetti, Licia Lugli, Isotta Guidotti, Maria Federica Roversi, Elisa Della Casa Muttini, Marisa Pugliese, Natascia Bertoncelli, Eugenio Spaggiari, Alessandra Todeschini, Gina Ancora, Sara Grandi, Giancarlo Gargano, Claudio Gallo, Mario Motta, Piero Catenazzi, Luigi Tommaso Corvaglia, Vittoria Paoletti, Agostina Solinas, Elisa Ballardini, Serafina Perrone, Sabrina Moretti, Marcello Stella, Alberto Berardi, Fabrizio Ferrari

**Affiliations:** ^1^ Neonatal Intensive Care Unit University Hospital of Modena Modena Italy; ^2^ Psycology Unit University Hospital of Modena Modena Italy; ^3^ Department of Neuroradiology University Hospital of Modena Modena Italy; ^4^ Neonatal Intensive Care Unit Infermi Hospital of Rimini Rimini Italy; ^5^ Neonatal Intensive Care Unit Azienda Unità Sanitaria Locale‐IRCCS Reggio Emilia Italy; ^6^ Neonatal Intensive Care Unit Maggiore Hospital of Bologna Bologna Italy; ^7^ Neonatal Intensive Care Unit Sant'Orsola Malpighi University Hospital of Bologna Bologna Italy; ^8^ Neonatal Intensive Care Unit Sant'Anna Hospital of Ferrara Ferrara Italy; ^9^ Neonatal Intensive Care Unit University Hospital of Parma Parma Italy; ^10^ Neonatal Intensive Care Unit Bufalini Hospital of Cesena Cesena Italy

**Keywords:** brain cooling, brain MRI, cerebral palsy, hypoxic–ischemic encephalopathy, therapeutic hypothermia

## Abstract

**Aim:**

This study assessed neurodevelopmental outcomes in neonates with hypoxic‐ischemic encephalopathy (HIE) treated with therapeutic hypothermia (TH) outside randomised controlled trials (RCTs). It also aimed to identify predictors of outcomes and evaluate TH practices across centres.

**Methods:**

A prospective, area‐based observational study was conducted in eight Italian NICUs (2016–2021), including neonates treated with TH for any grade of HIE. A 2‐year neurodevelopmental follow‐up was performed. Severe functional disability (SFD) was defined as cerebral palsy (Gross Motor Function Classification Level > 2), cognitive score < 2 SD, bilateral blindness/deafness, or epilepsy. Demographic, clinical and MRI data were analysed.

**Results:**

Among 283 cooled infants, 11 (3.8%) died and 272 (96.2%) survived. HIE severity was mild (14.0%), moderate (76.1%) and severe (9.9%). Follow‐up data were available for 232 (85.3%) survivors, with SFD diagnosed in 27 (11.6%). No infants with mild HIE developed SFD. Severe MRI anomalies were found in 51.9% of SFD cases, while 90.7% of non‐SFD children had normal findings. cEEG/aEEG‐confirmed seizures (OR = 12.9, CI 3.5–65.0) and severe MRI anomalies (OR = 0.24, CI 0.13–0.44) were strong SFD predictors (AUC = 0.95).

**Conclusion:**

Mortality and SFD rates were lower than in RCTs. Seizures and severe MRI anomalies predicted poor outcomes. Further RCTs are needed to refine treatment criteria.

**Abbreviations**
HIE: hypoxic–ischemic encephalopathy
IQR: interquartile range
OR: odds ratio
RCTs: randomised controlled trials
SFD: severe functional disability
TH: therapeutic hypothermia

**Summary**

- This study investigated neurodevelopmental outcomes at 2 years of age in infants treated with therapeutic hypothermia (TH) outside randomised clinical trials.
- Rates of mortality and severe disability were low, whereas seizures and severe MRI abnormalities were key predictors.
- The study highlights the growing use of TH in mild HIE and the need for trials to assess its effect in low‐risk infants.

## 1 Introduction

Therapeutic hypothermia (TH) has been a standard treatment since 2010 [1, 2], significantly reducing mortality and severe neurodevelopmental impairment in neonates with moderate‐to‐severe hypoxic–ischemic encephalopathy (HIE). Despite widespread adoption, variability exists in eligibility criteria for TH across international guidelines and consensus statements [3]. In addition, over time, increasing clinician confidence has expanded TH use to include neonates with mild HIE [4, 5, 6, 7]. Although these premises emphasise the need for assessing real‐world outcomes, only a few multicentre studies [8, 9, 10, 11, 12], mostly dated, have examined long‐term neurodevelopmental outcomes in children treated with TH in routine clinical practice. Recently, it has been reported that only 10 registries worldwide included variables on long‐term neurological outcomes in neonates with HIE [13].

This study aimed to assess the rate of severe functional disability (SFD) at 2 years of age in neonates treated with TH in an Italian network. Secondary objectives included identifying clinical and instrumental predictors of outcomes and evaluating TH practices across centres.

## 2 Materials and Methods

This prospective, observational study was conducted by the Neuronat Network, comprising eight level 3 NICUs in a northern region of Italy (Emilia‐Romagna), with approximately 35 000 live births annually. Established in 2015, the Network developed shared study protocols aligned with Italian Recommendations for TH [14]. Data collection was based on a regional registry using a customised REDCap form, including clinical, neuroimaging and neurodevelopmental outcomes. Each centre's principal neonatologist entered anonymous data derived from medical records, with a coordinating centre ensuring data completeness and accuracy through periodic communication and on‐site support when needed. Patient outcomes were reviewed during regular network meetings.

### 2.1 Study Population

This study included all infants born between 1 January 2016 and 31 December 2021, who underwent TH and completed neurodevelopmental follow‐up at 24 months of age. Since 2012, Italy has implemented national TH recommendations [14] based on clinical and instrumental criteria. Eligibility criteria for TH included: (1) intrapartum asphyxia (10‐min Apgar score ≤ 5, prolonged resuscitation, or severe acidosis [pH ≤ 7.0 or base deficit ≥ 12 mmol/L within 60 min of birth]); (2) moderate‐to‐severe neonatal encephalopathy (based on Shalak and Shankaran criteria) [15, 16] assessed within 6 h; and (3) moderate‐to‐severe anomalies on conventional EEG (cEEG) or amplitude‐integrated EEG (aEEG). In cases where aEEG was normal despite the presence of perinatal asphyxia criteria, the recommendations specified that priority should be given to neurological examination findings.

TH was performed with a target rectal temperature of 33.5°C. Passive cooling with temperature monitoring was provided during transport from spoke to hub centres. TH began within 6 h of birth, lasted 72 h, and was followed by gradual rewarming at 0.5°C/h. aEEG monitoring (with cEEG in one centre) was performed continuously from admission to the end of rewarming. Details regarding TH timing and complications were recorded.

The classification of HIE severity (mild, moderate and severe) used in the study was based solely on clinical assessment (based on Shalak and Shankaran criteria) [15, 16], and it was consistent with the neurological severity criteria defined in the national guidelines (see Appendix A for further details) [14]. Both neurological and neurophysiological assessments and grading were performed within the first hours of life by neonatologists with specific expertise in neonatal cEEG/aEEG interpretation, as part of the routine clinical evaluation for TH eligibility.

The cEEG findings were classified as follows: normal (continuous background pattern with normal amplitude, symmetric activity and normal physiological features such as anterior slow waves), mild abnormalities (continuous background pattern with slightly abnormal activity such as mild asymmetry, mild voltage depression, or poorly defined sleep–wake cycling), moderate abnormalities (discontinuous activity with interburst intervals of < 10 s, absence of clear sleep–wake cycling, or marked asymmetry/asynchrony), and severe abnormalities (discontinuous activity with interburst intervals of more than 10 s, severe attenuation of background activity or inactive trace, with absence of sleep–wake cycling) [17].

The aEEG findings were classified as follows: normal (lower margin > 5 μV and upper margin > 10 μV), moderate (lower margin ≤ 5 μV and upper margin > 10 μV) and severe abnormalities (lower margin < 5 μV and upper margin < 10 μV) [18, 19].

For the purpose of analysis, cEEG and aEEG findings classified as ‘normal’ and ‘mildly abnormal’ were grouped together into a single category, due to the absence of a distinct ‘mildly abnormal’ category in standard aEEG classifications.

Electrographic seizure was defined as a sudden repetitive, stereotyped discharge of minimum 10 s' duration on ≥ 1 cEEG channels with evolving frequency, amplitude and morphology [17]. At the aEEG, the seizure activity was defined as an abrupt rise in amplitude [18] (all suspected events were systematically verified by reviewing the corresponding raw EEG trace).

Seizure types were divided into two main subgroups: electrographic only and electroclinical.

The clinical presentation included motor seizures (such as automatisms, clonic movements, epileptic spasms, myoclonic and tonic seizures) and non‐motor findings [20]. The data on reported seizures in this study refer to episodes evaluated at the time of recording through electroclinical observation and documented in the clinical records.

### 2.2 Neurodevelopmental Follow‐Up

Neurodevelopmental assessment at 24 months of age was conducted by a developmental psychologist and neonatologist at each centre, with the participation of a child physiotherapist in most cases. Evaluations included the Amiel‐Tison neurological assessment [21] and either the Griffiths Mental Developmental Scales or the Bayley Scales of Infant and Toddler Development (BSDI III) [22, 23] depending on the local protocol. The Griffiths Mental Developmental Scales provided a General Development Quotient, while the Bayley Scales of Infant and Toddler Development measured standardised cognitive composite scores, with abnormality defined as < 2 SD below the mean. SFD was defined as at least one of the following: cerebral palsy (Gross Motor Function Classification level > 2) [24], mental development and cognitive score < 2 SD, bilateral blindness, bilateral deafness requiring hearing aids or implants, or epilepsy.

### 2.3 Brain MRI Findings

All infants underwent brain MRI under normothermic conditions between 4 and 10 days of life, using conventional and diffusion‐weighted sequences optimised for neonates. Experienced neuroradiologists assessed the images. Based on the MRI reports, findings were classified into five categories according to the classification proposed by Rutherford et al., which relies on conventional MRI sequences (see Appendix A for details) [25]. We chose this classification due to its previous validation [25] and our group's prior experience with it [26]. Since the first MRI could have been performed very early, often outside the optimal window for conventional sequences, when available, the latest MRI acquired within the first month of life was used for scoring.

### 2.4 Statistics

Infant characteristics were summarised using descriptive statistics. Continuous variables were expressed as median and interquartile range (IQR), and categorical variables as frequencies and percentages. Differences between groups were assessed using Pearson's *χ* ^2^ test for categorical variables and the Mann–Whitney *U* test for continuous variables. The mismatch between severity of HIE assessed clinically and cEEG/aEEG abnormalities was calculated together with Cohen's kappa coefficient. Uni‐ and multivariate logistic regression analyses were performed to identify factors associated with SFD (gestational age, sex, place of birth, type of birth, perinatal data, severity of HIE and of cEEG/aEEG anomalies, brain MRI findings). A stepwise selection strategy was used for including variables in the multivariate model. Multicollinearity was assessed using correlation coefficients and variance inflation factors. Odds ratios (OR) with 95% confidence intervals were calculated. Statistical significance was set at *p* < 0.05. Data were analysed using STATA (version 13.0). Since the study was based on a regional registry, sample size calculation was not planned.

### 2.5 Ethics Statement

The study was performed in accordance with the ethical standards of the responsible committee on human experimentation and with the Helsinki Declaration of 1975, as revised in 1983. The local Ethics Committee approved the project on the 14 of October 2015 (protocol number 221/15). Parents or guardians were provided with a written patient information leaflet prior to consent and enrolment in the research study.

## 3 Results

Based on data from the Regional Health Agency [27], 185.853 neonates were delivered during the study period. Among them, 283 (1.5/1000 live births) were cooled, with 11 infants (3.8%) dying within the first month of life (no cases were due to the redirection of care). Excluding cooled newborns with mild encephalopathy (*N* = 38), the mortality rate was 4.5% (11/245).

Of the remaining 272 infants, 37 (13.6%) did not complete the follow‐up, and 3 (1.1%) were excluded (because they were healthy at birth and suffered from sudden unexpected post‐natal collapse, *n* = 2; because TH was discontinued before 72 h, *n* = 1). The remaining 232 (85.3%) children completed the follow‐up and are the subject of the study.

Statistical differences between groups with and without follow‐up were observed for maternal race (higher prevalence of Black or Asian mothers among children without follow‐up, 14/37 [37.8%] vs. those with a follow‐up 29/232 [12.5%], *p* < 0.001) and caesarean delivery frequency (more common in children without follow‐up, 22/37 [59.5%] vs. those with a follow‐up, 87/232 [37.5%], *p* = 0.012).

Regarding infants who completed follow‐up, rates of mild, moderate and severe hypoxic–ischemic encephalopathy (HIE) were 30 (12.9%), 180 (77.6%) and 22 (9.5%), respectively.

Among 30 infants with mild HIE (no criterion 2 of recommendations), 21 met only criterion 1 (low Apgar scores, prolonged resuscitation and abnormal blood gas values) 3 met only criterion 3 (abnormal cEEG/aEEG findings) and 6 met both eligibility criteria (1 and 3).

Sentinel events occurred in 28.4% of infants (most commonly placental abruption and uterine rupture). Cardiotocographic anomalies were present in 36.2% of cases. Caesarean delivery was associated with cardiotocographic anomalies (64/87 [73.6%] vs. 63/144 [43.8%], *p* < 0.001) and more severe HIE (*p* = 0.007).

Most infants (98/232 [43%]) had moderate cEEG/aEEG abnormalities. A mismatch between the severity of HIE assessed clinically and cEEG/aEEG abnormalities was observed in 109/228 cases (48.7%). Mismatch rates were 9/29 (31.0%), 93/178 (52.2%) and 7/21 (33.3%) for mild, moderate and severe HIE grades, respectively. cEEG/aEEG overestimated the clinical grading of encephalopathy in 34/207 cases (16.4%), while clinical examination overestimated cEEG/aEEG severity in 75/188 cases (39.9%). When stratified by type of cEEG/aEEG severity, mismatches were identified in 25/60 (41.7%) and 84/168 (50%) of infants undergoing cEEG or aEEG, respectively. Agreement was slight in the overall population (*κ* = 0.20, 95% CI: 0.11–0.30) but mildly higher in the cEEG group (*κ* = 0.23, 95% CI: 0.05–0.42) as compared with the aEEG group (*κ* = 0.19, 95% CI: 0.09–0.30).

Seizures during TH were observed in 67 of 232 (28.9%) infants (only electrical, *n* = 16; both, electrical and clinical, *n* = 48; unspecified, *n* = 3). Seizures were less common among infants without SFD (no SFD, 42/203 [20.7%] vs. SFD, 25/27 [92.6%], *p* < 0.001). The median age at onset of seizures was 12 h (IQR 6–24) without significant differences between infants with or without SFD. One infant developed seizures during re‐warming (78 h after birth). Phenobarbital and phenytoin were the most used anticonvulsant drugs (68.7% and 49.3% respectively); a few infants were administered benzodiazepines, levetiracetam, or vitamins as additional therapy (31.3%, 4.4% and 3.0% of cases, respectively). No culture‐proven sepsis or meningitis cases were reported. Further details regarding characteristics in 232 infants with complete follow‐up and the comparison between infants with or without SFD at 24 months of age are reported in Table 1.

**TABLE 1:** Demographics and clinical characteristics in 232 infants with complete follow‐up, and comparison between infants with and without SFD at 24 months of age.

Variables	All infants (*N* = 232)	Missing	Infants with SFD (*N* = 27)	Infants without SFD (*N* = 205)	*p*
Gestational age, weeks	39.9 (38.3–40.7)	—	39.3 (38.3–40.3)	40 (38.4–40.7)	0.540
Birth weight, g	3287 (2885–3692)	—	3200 (2798–3750)	3300 (2938–3697)	0.535
Male sex	136 (58.6)	—	16 (59.3)	120 (58.5)	0.943
Twins	7 (3.0)	2	1 (3.7%)	6 (3.0%)	0.832
Non hispanic ethnicity	221 (97.3)	5	26 (96.3)	195 (97.5)	0.714
Outborn^a^	75 (32.3)	—	11 (40.7)	64 (31.2)	0.320
Sentinel events^b^	66 (28.4)	—	10 (37.0)	56 (27.3)	0.293
Maternal complications during pregnancy^c^	87 (37.5)	——	12 (46.2)	75 (36.9)	0.362
PROM	39 (17.4)	—	3 (11.5)	36 (18.2)	0.401
Caesarean section	87 (37.6)	1	17 (63.0)	70 (34.3)	0.004
10 min apgar score, median	7 (6–8)	21	6 (4–6)	7 (6–8)	0.004
pH, median^d^	6.97 (6.84–7.01)	15	6.8 (6.8–6.9)	6.9 (6.9–7.1)	< 0.001
BE, median^d^	−16.1 (−29.1;‐13.3)	8	−21.1 (−25.3;−16.1)	−16 (−19;‐13)	< 0.001
Resuscitation at birth					
Intubation	109 (47.0)		17 (63.0)	92 (44.9)	0.077
Chest compression	49 (21.1)		9 (33.3)	40 (19.5)	0.098
Epinephrine	27 (11.6)	—	3 (11.1)	24 (11.7)	0.928

*Note:* Data are presented as median (interquartile range) or number (%). Adverse events include bradycardia (*N* = 33, 14.5%), persistent pulmonary hypertension (*N* = 23, 10.1%), thrombocytopenia (*N* = 18, 7.9%), adiponecrosis (*N* = 8, 3.5%), pulmonary haemorrhage (*N* = 4, 1.8%), gastrointestinal bleeding (*N* = 1, 0.4%).

Abbreviations: PROM, prolonged rupture of membrane; SFD, severe functional disability.

^a^ No differences in the severity of HIE were observed between inborn and outborn infants.

^b^ Sentinel events occurred in 232 infants: placenta abruption (*N* = 27, 11.6%), uterine rupture (*N* = 8, 3.4%), funiculus prolapsus (*N* = 6, 2.6%), amniotic embolism (*N* = 1, 0.4%), acute anaemia (*N* = 1, 0.4%), shoulder dystocia (*N* = 7, 3.0%), maternal collapse (*N* = 3, 1.3%), unspecified (*N* = 5, 2.2%).

^c^ Diabetes (*n* = 35), hypertension (*n* = 17), unspecified maternal complications (*n* = 43).

^d^ Collected from umbilical cord within 60 min from birth.

### 3.1 Neurodevelopmental Outcome at 24 Months of Age, Cerebral MRI Findings

SFD was diagnosed in 11.6% (27/232) of children (in 13.4% [27/202] among children with moderate‐to‐severe HIE only), and cerebral palsy (CP) was the most common impaired outcome.

None of the infants with mild HIE developed SFD, while 9.4% of those with moderate and 45.5% of those with severe HIE developed SFD. Severe HIE increased the risk of SFD nearly eightfold compared to moderate HIE (OR 7.9, CI 3.0–21.2, *p* < 0.001).

**FIGURE 1:**
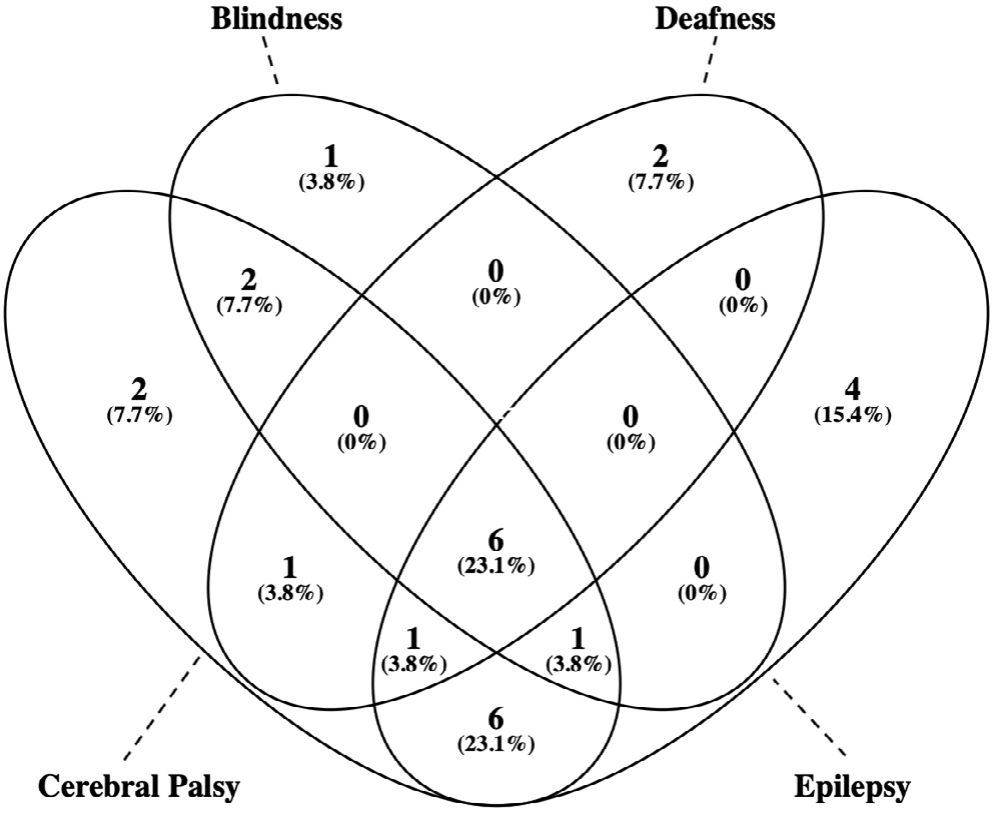
Overlapping of neurodevelopmental impairments in children with severe functional disability. Two children had severe cognitive impairment (in one case associated with epilepsy).

As reported in Table 1 and in Table 2, infants with SFD were more likely to have a caesarean delivery, a lower 10‐min Apgar score, a lower pH and base deficit, more severe HIE, cEEG/aEEG anomalies and severe brain MRI patterns. CP was the most common neurological sequela, affecting 21 (9.1%) of 232 children. Among them, 18 (85.7%) had Gross Motor Function Classification level > 2. Some children had additional disabilities, mostly associated with cerebral palsy. Figure 1 shows the overlapping of disabilities in children with SFD.

**TABLE 2:** Neurological, neurophysiological and neuroradiological characteristics in 232 infants with complete follow‐up, and comparison between infants with and without SFD at 24 months of age.

Variables	All infants (*N* = 232)	Missing	Infants with SFD (*N* = 27)	Infants without SFD (*N* = 205)	*p*
HIE severity					
Mild	30 (12.9)	—	0 (0)	30 (14.6)	< 0.001
Moderate	180 (77.6)		17 (63.0)	163 (79.5)	
Severe	22 (9.5)		10 (37.0)	12 (5.9)	
cEEG/aEEG abnormality on admission^b^					
Normal/mild	90 (39.5)	4	4 (16.0)	86 (42.4)	< 0.001
Moderate	98 (43.0)		5 (20.0)	93 (45.8)	
Severe	40 (17.5)		16 (64.0)	24 (11.8)	
MRI patterns					
Pattern 1	15 (6.5)	—	14 (51.9)	0 (0)	< 0.001
Pattern 2	5 (2.2)		1 (3.7)	2 (1.0)	
Pattern 3	10 (4.3)		4 (14.8)	7 (3.4)	
Pattern 4	11 (4.7)		5 (18.5)	10 (4.9)	
Pattern 5	191 (82.3)		3 (11.1)	186 (90.7)	
Two MRI performed^a^	119 (51.3)	—	25 (92.6)	94 (45.9)	< 0.001

*Note:* Data are presented as median (interquartile range) or number (%).

Abbreviations: cEEG/aEEG, conventional electroencephalography/amplitude electroencephalography; HIE, hypoxic–ischemic encephalopathy; MRI, magnetic resonance imaging; SFD, severe functional disability.

^a^ Median age at the time the last MRI was performed was 41.5 days.

^b^ The assessment was performed within the first 6 h of life, prior to the initiation of therapeutic hypothermia.

Epilepsy was the most common impairment after CP, affecting 18 infants (7.7%) with HIE (mild HIE, 0%; moderate HIE, 6.6%; and severe HIE, 27.3%). Epilepsy developed within the first 6 months of life in 17 out of 18 children, and 15 children (83.3%) had associated comorbidities. At univariate analysis, epilepsy was significantly associated with a lower pH (OR 0.001, CI < 0.001–0.07, *p* 0.001) or bases excess at birth (OR 0.83, CI 0.74–0.92, *p* 0.001), severe HIE (OR 6.96, CI 2.43–19.90, *p* < 0.001), severe cEEG/aEEG abnormalities (OR 3.49, CI 1.65–7.40, *p* 0.001), seizures during TH (OR 62.06, CI 8.03–479.65, *p* < 0.001) and brain MRI abnormalities (OR 0.19, CI 0.11–0.32, *p* < 0.001).

Cognitive assessments (available for 64.3% of children) (details in Table 3) showed that most children had scores > −1 SD. Seven children were diagnosed with an autism spectrum disorder.

**TABLE 3:** Neurodevelopmental impairment among 232 children undergoing TH who completed the follow‐up.

Variables	Children with a compete follow up (*n* = 232)
Cerebral palsy, *N* (%)^a^	21 (9.1)
Blindness, *N* (%)	10 (4.3)
Deafness, *N* (%)	10 (4.3)
Epilepsy, *N* (%)	18 (7.7)
Cognitive scores^b^	
GMDS‐R	
Global quotient, median (IQR)	108 (101–114)
Locomotor quotient, median (IQR)	98 (98–107)
Personal and social quotient, median (IQR)	106 (99–119)
Hearing and language quotient, median (IQR)	107 (98–113)
Eye and hand quotient, median (IQR)	112 (107–118)
Performance quotient, median (IQR)	106 (100–117)
BSDI‐III	
Cognitive composite score, median (IQR)	100 (90–105)
Motor composite score, median (IQR)	94 (85–100)
Language composite score, median (IQR)	91 (83–97)
GMDS‐R global quotient/BSDI‐III cognitive compositive score	
Score < 70, *N* (%)	2/137 (1.5)
Score 70–85, *N* (%)	10/137 (7.3)
Score > 85, *N* (%)	125/137 (91.2)
Severe functional disability, *N* (%)	27 (11.6)

Abbreviations: BSDI‐III, Bayley Scales of Infant and Toddler Development; GMDS‐R, Griffiths Mental Developmental Scales; GQ, General Development Quotient; TH, therapeutic hypothermia.

^a^ Tetraplegia *n* = 17, diplegia *n* = 3, hemiplegia *n* = 1; Gross motor function grade 2, *n* = 3, grade 3 *n* = 1, grade 4, *n* = 3, grade 5, *n* = 14.

^b^ Nineteen children with tetra paresis or blindness were excluded from cognitive evaluation; 44/137 (32.1%) of children underwent Griffiths Mental Developmental Scales evaluation, and 93/137 (67.9%) underwent Bayley Scales of Infant and Toddler Development evaluation.

Regarding brain MRI, children with SFD were more frequently subjected to two neuroimaging examinations. Among them, 51.9% exhibited the most severe grey matter anomalies (pattern 1), whereas 90.7% of children without SFD had normal findings (pattern 5).

Severe HIE was associated with more severe brain lesions on MRI (patterns 1 and 2), which were never seen in mild HIE but were present in 5% of moderate and 36.4% of severe HIE cases. Conversely, the less severe MRI pattern (pattern 5) was more common in mild HIE (100%) and less frequent in moderate (83.3%) or severe HIE (40.9%).

### 3.2 Early Predictors of Severe Functional Disability at 24 Months of Age

Univariate logistic regression analysis found multiple factors associated with SFD (Table 4). Values of area under the ROC curve of clinical grade of HIE and of cEEG/aEEG abnormalities were 0.70 and 0.76 respectively, without significant difference (*p* = 0.207).

**TABLE 4:** Univariate and multivariate analyses of factors associated with severe functional disability.

	Univariate analysis			Multivariate analysis		
	OR	95% CI	*p*	OR	95% CI	*p*
Gestational age at birth	0.972	0.77–1.22	0.813			
Sex	0.970	0.42–2.19	0.943			
Outborn	1.514	0.66–3.44	0.323			
Caesarean section	3.254	1.41–7.48	0.005			
Presence of sentinel event	1.964	0.81–4.73	0.133			
Apgar 10th minute	0.747	0.60–0.92	0.008			
Intubation during resuscitation	2.088	0.91–4.77	0.081			
Timing to start TH	0.768	0.60–0.97	0.032			
BE	0.844	0.77–0.92	< 0.001			
Grade of HIE^a^	8.875	3.53–22.30	< 0.001			
EEG abnormalities at admission^b^	5.034	2.53–10.01	< 0.001			
Presence of seizures during TH	47.916	10.9–210.44	< 0.001	12.9	2.56–65.02	0.002
Cerebral MRI patterns	0.184	0.10–0.30	< 0.001	0.248	0.13–0.44	< 0.001

Abbreviation: BE, base excess.

^a^ Grade of HIE during the first 6 h after birth.

^b^ cEEG/aEEG.

In multivariate analysis, SFD remained associated with seizures during TH and brain MRI patterns.

**FIGURE 2:**
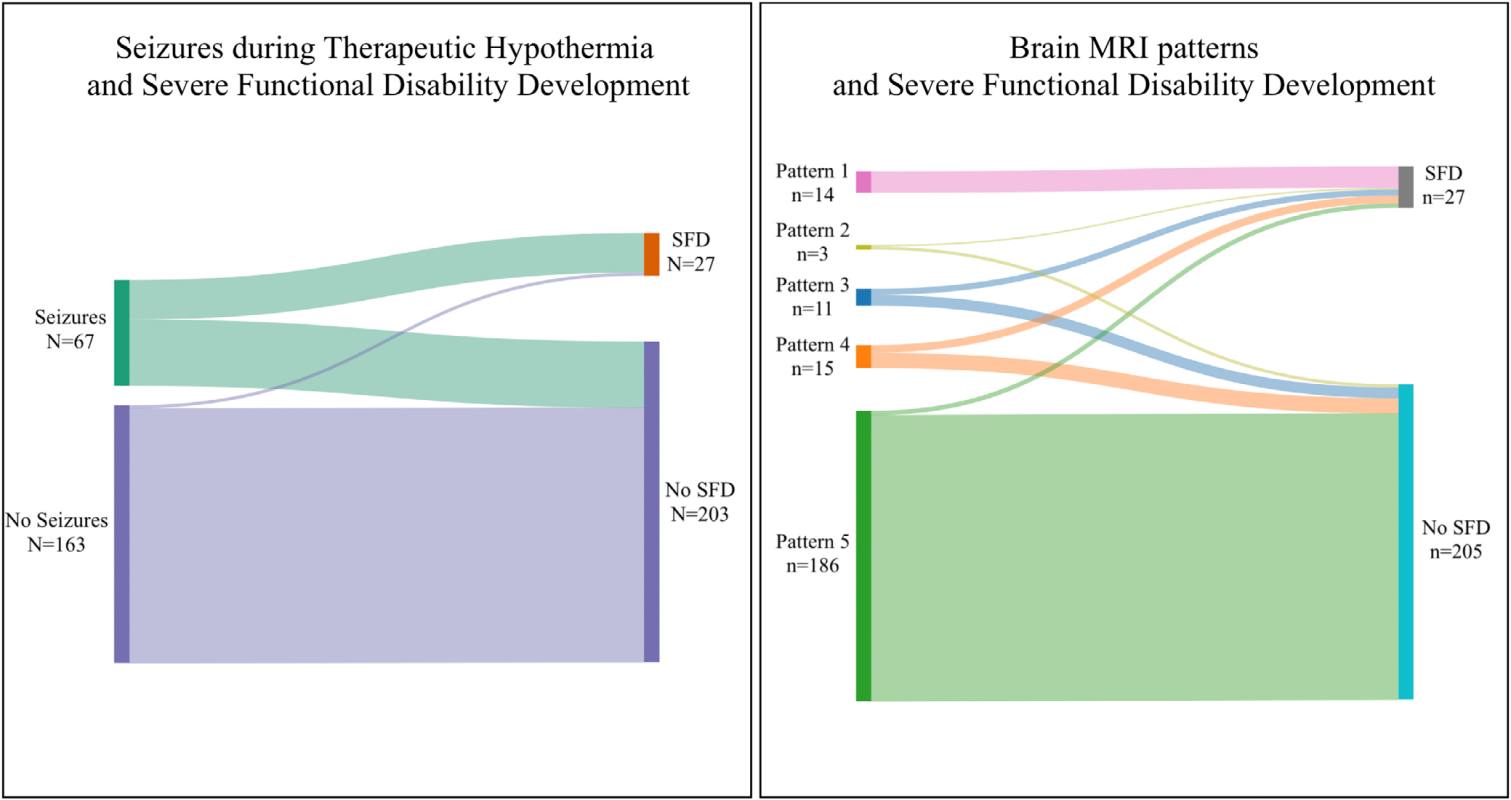
Sankey diagram of SFD according to the presence of seizures during TH and the five different patterns of brain MRI. SFD, severe functional disability.

The model had a sensitivity of 70.4%, specificity of 98.5%, positive predictive value of 86.4%, negative predictive value of 96.2%, and an area under the ROC curve of 0.95. Sankey diagrams illustrate outcomes (SFD of non‐SFD) based on seizures and MRI patterns (Figure 2).

### 3.3 Therapeutic Hypothermia Management and Adverse Effects

**FIGURE 3:**
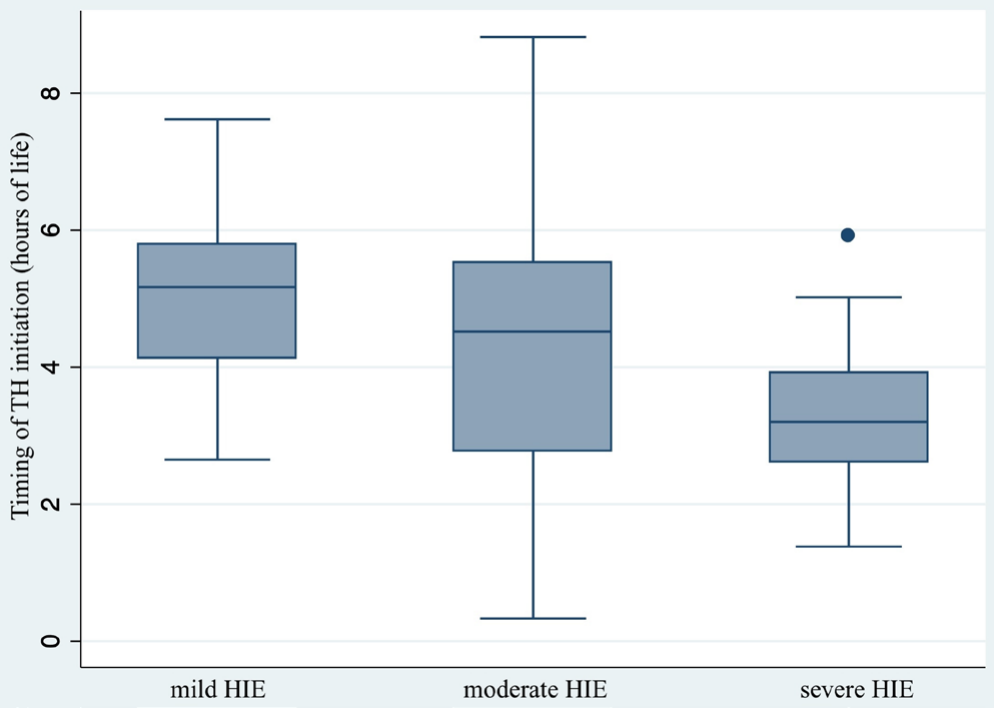
Time to initiation of TH according to the severity of HIE. HIE, hypoxic–ischemic encephalopathy; TH, therapeutic hypothermia. Median time to initiation of TH in infants with mild (5.2 h, IQR 4.1–5.8), moderate (4.5 h, IQR 2.8–5.5) or severe HIE (3.2 h, IQR 2.6–3.9), *p* = 0.001.

The median duration of TH was 72 h, with TH starting at a median of 4.3 h of life. TH was initiated earlier in inborn compared to outborn infants (3.9 vs. 5.5 h, *p* < 0.001). Time to initiation (Figure 3) was significantly delayed in HIE of lower severity. TH was started after 6 h in 11.9% of cases (median 6.7 h, IQR 6.25–7.2).

All newborns were given analgosedation with fentanyl, whereas benzodiazepines were administered in 29.5% of cases. Adverse events occurred in 58 of 232 (25.0%) infants: the most common adverse event was bradycardia (14.5%), followed by pulmonary hypertension (10.1%) (mostly considered part of the medical history rather than consequent to TH). Details regarding adverse events are reported in the footnote of Table 1.

## 4 Discussion

This is the first Italian prospective area‐based observational study evaluating 2‐year neurodevelopmental outcomes, early predictors of outcomes, and TH practices in children with HIE. Differently from some early studies, the current study also provides valuable brain MRI findings [8, 9, 10, 11, 12] and offers updated insights.

Overall mortality rates were low (3.8%), aligning with the lower range of observational studies (2.7%–32%) [8, 9, 10, 11, 12, 28] and significantly below those reported in RCTs (20%–38%) [1, 2].

The SFD rates were 11.6% (children with any degree of HIE) and 13.4% (children with moderate‐to‐severe HIE), and CP was the most common impaired outcome. These rates are significantly lower than those reported in RCTs (up to 60%) [1, 2] and most observational studies (20%–30%) [8, 9, 10, 12]. Possible explanations include the routine use of cEEG/aEEG in candidate selection, improved perinatal care, and the network's expertise in TH application [8, 28]. Despite the inclusion of mild HIE cases potentially inflating favourable outcomes, SFD rates remained low even in moderate‐to‐severe HIE cases. These findings, combined with low mortality and no trend toward redirection of care, would reflect the strengths of the regional healthcare system and obstetric care quality.

Predicting adverse neurodevelopmental outcomes is crucial for parental counseling and early identification of children who may benefit from early interventions [29, 30, 31, 32, 33]. In this study, early markers such as seizures during TH and the severity of brain lesions on MRI were highly predictive of SFD. Predominant grey matter lesions on MRI were associated with the highest SFD risk, consistent with findings from earlier studies and those conducted during the TH era [10, 11, 25, 26, 29, 31, 34, 35, 36]. Recently, general movements and cEEG findings have been identified as additional predictors of SFD in infants undergoing TH [31, 32, 37]. However, data on general movements were unavailable in this study due to the lack of routine collection, and cEEG use was limited to a single centre.

Our findings confirm that infants with neonatal HIE are at higher risk of developing epilepsy later in life, more commonly within the first 6 months, with additional associated comorbidities. Key early predictors of the development of epilepsy are the severity of hypoxic–ischemic insult at birth, severe encephalopathy, severe cEEG/aEEG abnormalities, acute provoked seizures during TH, and severe brain lesions [20, 38].

The agreement between HIE severity and cEEG/aEEG abnormalities was slight (particularly in moderate cases), reflecting inherent difficulties of accurately classifying infants with HIE. Consistent with the existing literature [19, 39], cEEG showed better agreement with HIE than aEEG. cEEG is widely recognised as the gold standard for neurophysiological evaluation and prognostication, especially when continuous monitoring is performed, although its use is constrained by the need for specialised expertise [39]. While our neurophysiological stratification was limited to the early postnatal period and primarily focused on seizure monitoring within 72 h of life, it still provided valuable prognostic insights. The prognosis of SFD showed a higher area under the ROC curve for cEEG/aEEG severity with respect to HIE severity assessed clinically, although not significantly. cEEG/aEEG abnormalities and seizures further highlight the importance of both cEEG and aEEG combined with clinical evaluation in the assessment of HIE. To date, continuous monitoring through cEEG (or alternatively aEEG) is recommended before the initiation and during TH, until the rewarming phase [40].

Such efforts to accurately classify disease severity continue to represent a challenge for clinicians, and in clinical practice, deviations from TH guidelines are not uncommon [41, 42, 43]. In this study, TH was administered to neonates outside Italian recommendations, including those with a gestational age < 35 weeks (*n* = 9) and those treated more than 6 h after birth (*n* = 29).

Additionally, 12.9% of infants who received TH had mild HIE, aligning with rates reported in other studies (4%–23%) [9, 10, 41, 42, 43]. This trend has increased over time [4, 5, 6, 7] despite no guideline updates [41]. Contributing factors may include variability in inclusion criteria across RCTs [1] and international guidelines [3]. Factors such as sentinel events, resuscitation, metabolic status at birth and difficulties in accurately classifying HIE severity [3, 39, 44] can significantly impact decisions regarding TH in clinical practice [3, 33]. In this study, as expected [7, 9, 10, 41], no children with mild HIE developed SFD, reinforcing the notion that mild HIE is a low‐risk neurological category. Because we lack a control group, the favourable outcome observed after mild HIE cannot be conclusively attributed to TH, and in such cases, TH remains questionable; studies as the ongoing COOLPRIME study [45], could provide insights on this issue [34, 35].

Notably, in this study, neonates with mild HIE received TH later than those with moderate‐to‐severe HIE, reflecting clinicians' cautious approach in borderline cases. Since infants with moderate‐to‐severe HIE underwent TH earlier than those with mild HIE, it was not possible to accurately assess the effectiveness of TH in relation to the timing of its initiation [46].

In addition, the observation that treatment was initiated after 6 h in 11.9% of cases indicates that there are still areas for improvement in timely management.

This study has several limitations. First, we lack a control group for cases of mild HIE, limiting the ability to draw firm conclusions regarding the effects; consequently, our findings are primarily descriptive. Secondly, cognitive assessment at 24 months was sometimes missing, potentially leading to overestimation of favourable outcomes. However, HIE is rarely associated with isolated severe cognitive deficits [31]. Third, the 24‐month follow‐up period may be insufficient to detect more subtle neurodevelopmental impairments that often become apparent later in childhood [47, 48, 49]. Finally, brain MRI scoring was based on reports provided from each centre, and it relied on conventional rather than diffusion‐weighted imaging. These limitations suggest the need for further research in the neuroimaging field.

## 5 Conclusions

Mortality and overall SFD rates following cooling for HIE were lower than those in previous RCTs and cohort studies. Of the cooled infants, 12.9% had mild HIE, with none developing SFD. Seizures during TH and severe brain MRI abnormalities emerged as the most significant predictors of poor outcomes. These findings, provided from a multicentre, real‐world context, strengthen TH applicability in routine care and support informed clinical decision‐making beyond the research setting. Future RCTs could help identify new treatment indicators for neonates with lower HIE severity.

## Author Contributions

**Luca Bedetti:** methodology, formal analysis, investigation, data curation, writing – original draft, writing – review and editing, visualization. **Licia Lugli:** conceptualization, methodology, formal analysis, investigation, writing – review and editing, visualization, supervision, funding acquisition. **Isotta Guidotti:** conceptualization, methodology, investigation, formal analysis, data curation, writing – review and editing, visualization, supervision. **Maria Federica Roversi:** investigation, writing – review and editing, visualization. **Elisa Della Casa Muttini:** investigation, visualization, writing – review and editing. **Marisa Pugliese:** investigation, visualization, writing – review and editing. **Natascia Bertoncelli:** investigation, visualization, writing – review and editing. **Eugenio Spaggiari:** investigation, visualization, writing – review and editing. **Alessandra Todeschini:** investigation, writing – review and editing, visualization. **Gina Ancora:** methodology, investigation, writing – review and editing, visualization. **Sara Grandi:** investigation, visualization, writing – review and editing, data curation. **Giancarlo Gargano:** investigation, visualization, writing – review and editing. **Claudio Gallo:** investigation, visualization, writing – review and editing, data curation. **Mario Motta:** investigation, visualization, writing – review and editing. **Piero Catenazzi:** investigation, visualization, writing – review and editing, data curation. **Luigi Tommaso Corvaglia:** investigation, visualization, writing – review and editing. **Vittoria Paoletti:** investigation, visualization, writing – review and editing, data curation. **Agostina Solinas:** investigation, visualization, writing – review and editing. **Elisa Ballardini:** investigation, visualization, writing – review and editing, data curation. **Serafina Perrone:** investigation, visualization, writing – review and editing. **Sabrina Moretti:** investigation, visualization, writing – review and editing, data curation. **Marcello Stella:** investigation, visualization, writing – review and editing, data curation. **Alberto Berardi:** investigation, writing – original draft, writing – review and editing, visualization, supervision. **Fabrizio Ferrari:** conceptualization, methodology, formal analysis, investigation, writing – original draft, writing – review and editing, visualization, supervision, funding acquisition.

## Conflicts of Interest

The authors declare no conflicts of interest.

## Data Availability

The original contributions presented in the study are included in the article; further inquiries can be directed to the corresponding authors.

## Appendix A

**Neurological examination according to the modified Shalak and Shankaran criteria** [15, 16].

Encephalopathy was classified as: mild (hyperactivity, normal or increased tone, normal spontaneous movements and posture, tremors, exaggerated Moro reflex, no autonomic dysfunction), moderate (lethargy, reduced motility, distal flexion/complete extension, hypotonia, weak/incomplete primitive reflexes, myosis, bradycardia and periodic breathing) and severe (stupor or coma, decerebrated posture, absent motility, flaccid tone, absent reflexes, mydriatic/deviated/non‐reactive pupils, apnea). The prevalence of signs determined the grade of severity of encephalopathy; if neurological signs were evenly distributed among the various grades of severity, the grade of encephalopathy was defined based on the level of consciousness.

**Cerebral MRI patterns' definitions according to the classification of Rutherford et al.** [25].

- Pattern 1: basal ganglia and thalami lesions associated with severe white matter damage.
- Pattern 2: basal ganglia and thalami lesions with mild or moderate white matter changes.
- Pattern 3: isolated thalamic injury.
- Pattern 4: moderate white matter damage only.
- Pattern 5: mild white matter changes or normal findings.
